# A two-dimensional Cd^II^ coordination polymer: poly[di­aqua­[μ_3_-5,6-bis­(pyridin-2-yl)pyrazine-2,3-di­carboxyl­ato-κ^5^ *O* ^2^:*O* ^3^:*O* ^3^,*N* ^4^,*N* ^5^]cadmium]

**DOI:** 10.1107/S2056989016012858

**Published:** 2016-08-16

**Authors:** Monserrat Alfonso, Helen Stoeckli-Evans

**Affiliations:** a Institute of Chemistry, University of Neuchâtel, Av Bellevaux 51, CH-2000 Neuchâtel, Switzerland; b Institute of Physics, University of Neuchâtel, rue Emile-Argand 11, CH-2000 Neuchâtel, Switzerland

**Keywords:** crystal structure, cadmium(II), sevenfold coordination, penta­gonal bipyramid, two-dimensional coordination polymer, network, hydrogen bonding

## Abstract

The reaction of 5,6-bis­(pyridin-2-yl)pyrazine-2,3-di­carb­oxy­lic acid with cadmium dichloride leads to the formation of the title two-dimensional coordination polymer, [Cd(C_16_H_8_N_4_O_4_)(H_2_O)_2_]_*n*_. The metal atom is sevenfold coordinated by one pyrazine and one pyridine N atom, two water O atoms, and by two carboxyl­ate O atoms, one of which bridges two Cd^II^ atoms to form a Cd_2_O_2_ unit situated about a centre of inversion. Hence, the ligand coordinates to the cadmium atom in an *N*,*N*′,*O*-tridentate and an *O*-monodentate manner. Within the polymer network, there are a number of O—H⋯O hydrogen bonds present, involving the water mol­ecules and the carboxyl­ate O atoms. There are also C—H⋯N and C—H⋯O hydrogen bonds present. In the crystal, the polymer networks lie parallel to the *bc* plane. They are aligned back-to-back along the *a* axis with the non-coordinating pyridine rings directed into the space between the networks.

## Abstract 2

The reaction of cadmium dichloride with the ligand 5,6-bis­(pyridin-2-yl)pyrazine-2,3-di­carb­oxy­lic acid leads to the formation of a two-dimensional coordination polymer.

## Chemical context   

The crystal structure of the ligand 5,6-bis­(pyridin-2-yl)pyrazine-2,3-di­carb­oxy­lic acid (**H_2_L**) and the chloride, perchlorate and hexa­fluoro­phosphate salts, have been reported on previously (Alfonso *et al.*, 2001 ▸). Inter­estingly, the ligand crystallizes as a zwitterion in all four compounds. The reaction of **H_2_L** with CuBr_2_ (ratio 1:2) leads to the formation of a one-dimensional coordination polymer. On exposure to air, the compound loses the solvent of crystallization and four water mol­ecules, transforming into a two-dimensional coordination polymer (Neels *et al.*, 2003 ▸). In both cases, there are two crystallographically independent fivefold-coordinated copper atoms present and they all have almost perfect square-pyramidal geometry. Recently, we have reported on the crystal structures of the dimethyl and diethyl ester of the **H_2_L** ligand (Alfonso & Stoeckli-Evans, 2016*a* ▸). The reaction of the dimethyl ester of **H_2_L** with CdCl_2_ and HgCl_2_ leads to the formation of isotypic one-dimensional coordination polymers (Alfonso & Stoeckli-Evans, 2016*b* ▸). There the ligand coordin­ates to the metal atom *via* the pyridine N atoms, and they have *M*N_2_Cl_2_ fourfold bis­phenoidal coordination geometry.

## Structural commentary   

**Figure 1:**
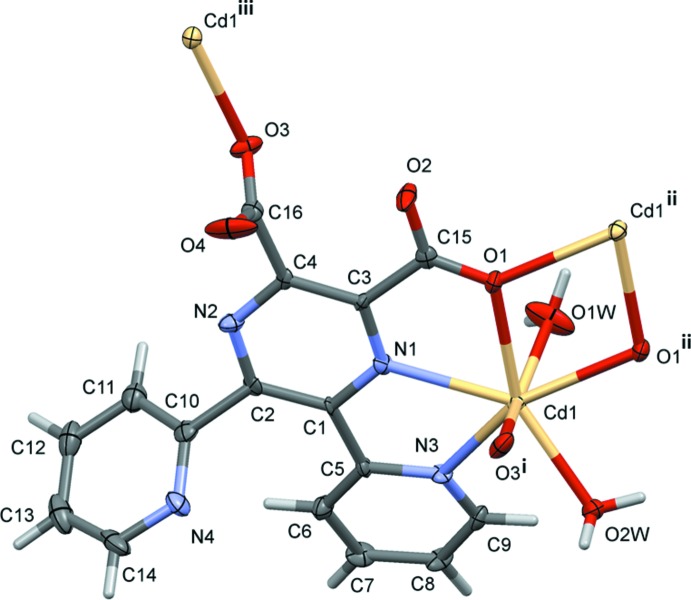
A view of the mol­ecular structure of the title coordination polymer, showing the atom labelling [symmetry codes: (i) *x*, −*y* + 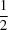, *z* − 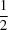; (ii) −*x* + 1, −*y* + 1, −*z* + 1; (iii) *x*, −*y* + 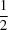, *z* + 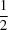]. Displacement ellipsoids are drawn at the 50% probability level.

The reaction of 5,6-bis­(pyridin-2-yl)pyrazine-2,3-di­carb­oxy­lic acid with cadmium dichloride leads to the formation of the title two-dimensional coordination polymer (Fig. 1 ▸). Here the metal atom is sevenfold coordinated by one pyrazine N atom (N1), one pyridine N atom (N3) and two water O atoms (O1*W* and O2*W*), and by two carboxyl­ate O atoms (O1 and O3). Atom O1 bridges two cadmium atoms to form a Cd_2_O_2_ unit situated about a centre of inversion; the Cd1⋯Cd1^ii^ distance is 3.8753 (8) Å, while the Cd—O1 and Cd—O1^ii^ bonds are, respectively, 2.371 (4) and 2.427 (4) Å, and the Cd1—O1⋯Cd1^ii^ and O1—Cd⋯O1^ii^ bond angles are 107.74 (13) and 72.26 (13)°, respectively. As can be seen in Fig. 1 ▸, the ligand coordinates to the cadmium atom in a tridentate (*N*,*N*,O) and a monodentate manner (*O*). It can be seen from the carboxyl­ate C—O bond lengths [C15—O1 and C15—O2 are 1.255 (6) and 1.253 (6) Å, respectively, while C16—O3 and C16—O4 are 1.258 (6) and 1.227 (6) Å, respectively] that the negative charge is distributed over the O–C–O group for the first, but located on atom O3 for the second.
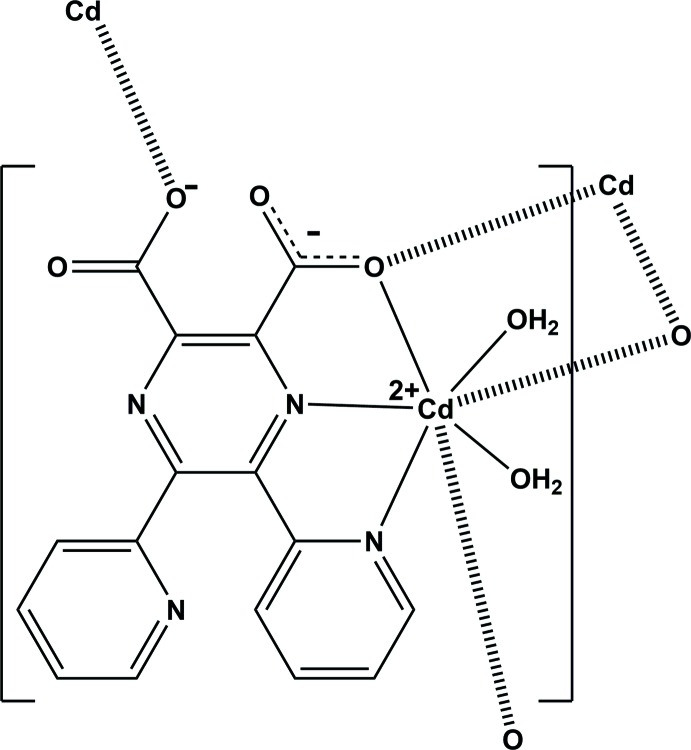


Selected bond lengths and angles involving atom Cd1 are given in Table 1 ▸. The Cd—N_pyrazine_ (Cd1—N1) and the Cd—N_pyridine_ (Cd1—N3) bond lengths are the same within 3 s.u.s. [2.418 (4) *cf.* 2.430 (4) Å]. The Cd—O_water_ bond lengths [2.301 (4) and 2.317 (3) Å] are shorter than the Cd—O_carboxyl­ate_ bond lengths [2.371 (4) and 2.377 (4) Å], while the bridging Cd1⋯O1^ii^ distance is the longest at 2.427 (4) Å. The geometry of the sevenfold-coordinated cadmium atom can best be described as a distorted penta­gonal bipyramid, with atoms O1,N1,N3,O2*W*,O1^ii^ in the basal plane and atoms O1*W*,O3^i^ in the apical positions with an O1*W*—Cd1—O3^i^ bond angle of 157.41 (15)° (Table 1 ▸).

The coordinated pyridine ring (N3/C5-C9) and the carboxyl­ate group (O1/O2/C15) are inclined to the pyrazine ring (r.m.s. deviation = 0.03 Å) by 16.9 (2) and 1.9 (6)°, respectively. The non-coordinating pyridine ring (N4/C10–C14) and the second coordinating carboxyl­ate group (O3/O4/C16) are inclined to the pyrazine ring by 60.2 (3) and 89.1 (11)°, respectively. The two pyridine rings are inclined to one another by 75.4 (3) °.

## Supra­molecular features   

**Figure 2:**
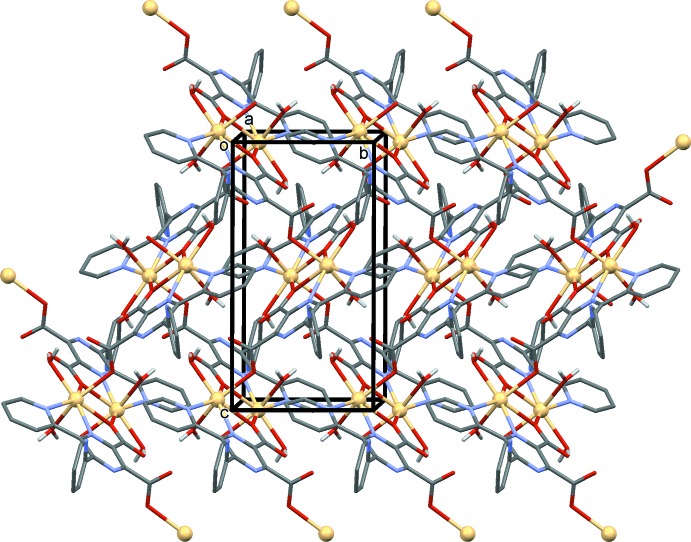
A view along the *a* axis of the title two-dimensional coordination polymer. The C-bound H atoms have been omitted for clarity.

**Figure 4:**
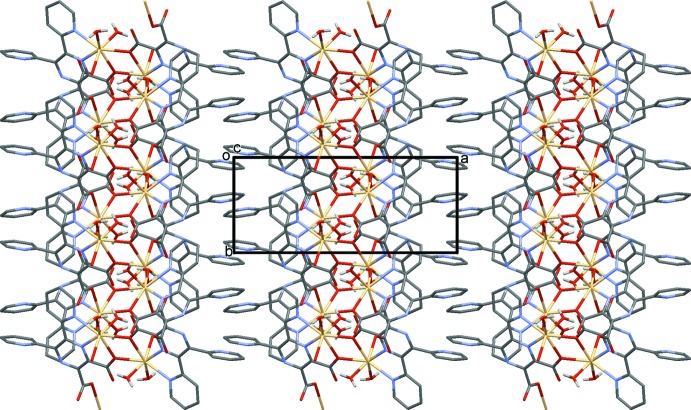
A view in projection down the *c* axis of the crystal packing of the title two-dimensional coordination polymer. The C-bound H atoms have been omitted for clarity.

**Figure 5:**
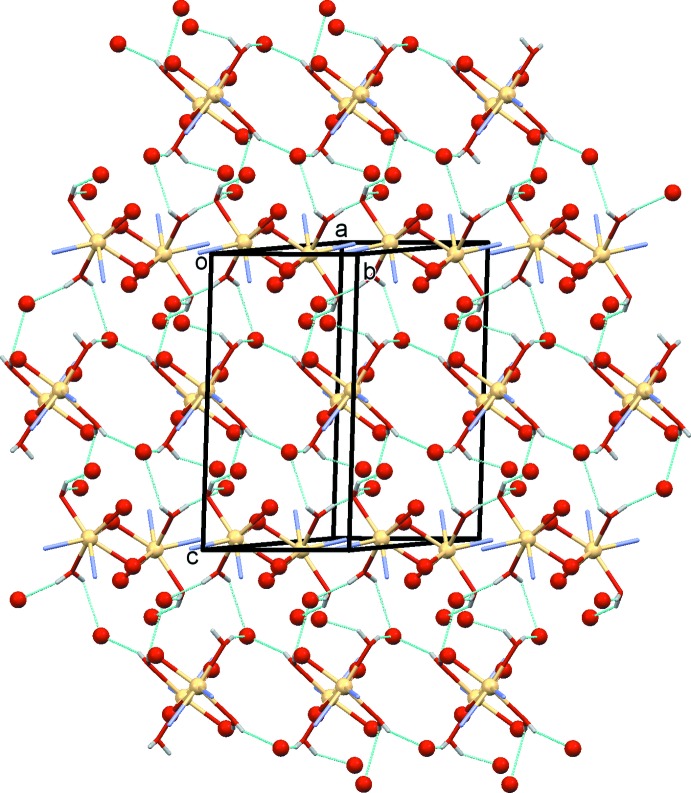
A view normal to plane (1
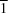
0) of the O—H⋯O hydrogen bonds (dashed lines; see Table 2 ▸) within the polymer network, involving the carboxyl­ate O atoms (red balls) and the coordinating water mol­ecules. The C atoms and C-bound H atoms of the ligand have been omitted for clarity.

In the crystal, the two-dimensional polymer networks lie parallel to the *bc* plane, as illustrated in Figs. 2 ▸ and 3 ▸. The networks are aligned back-to-back along the *a* axis, with the non-coordinating pyridine rings directed into the space between the networks (Fig. 4 ▸). Within the networks there are a number of O—H⋯O hydrogen bonds present, involving the water mol­ecules and the carboxyl­ate O atoms (Table 2 ▸ and Fig. 5 ▸). There are also C—H⋯O and C—H⋯N hydrogen bonds present within the network (Table 2 ▸).

## Database survey   

A search of the Cambridge Structural Database (CSD, Version 5.37, last update May 2016; Groom *et al.*, 2016 ▸) for the ligand **H_2_L** gave eight hits. All of these structures have been mentioned in the *Chemical context* above. A search for cadmium complexes with the Cd atom coordinated by two N atoms, two water mol­ecules and three O atoms, two of which are carboxyl­ate O atoms, gave seven hits. One of these compounds, catena-[(μ_2_-1,1′-(butane-1,4-di­yl)bis­(5,6-dimethyl-1*H*-benzimidazole)]bis­(μ_2_-pyridine-2,6-di­carboxyl­ato)tetra­aqua­dicadmium dihydrate) [CSD refcode: FAVHIV; Jiao *et al.*, 2012 ▸] has a Cd_2_O_2_ unit formed about an inversion centre as in the title compound. In FAVHIV, the Cd⋯Cd distance and the angles Cd—O⋯Cd and O—Cd⋯O are, respectively, 4.0408 (5) Å, and 111.05 (8) and 68.95 (7)°, compared to 3.8753 (8) Å, and 107.74 (13) and 72.26 (13) °, respectively, in the title compound. However, such an arrangement is extremely common for cadmium(II) complexes (over 600 hits in the CSD) and the bond lengths and angles vary enormously; for example the Cd⋯Cd distance varies from *ca* 3.0 to 4.3 Å, the Cd—O⋯Cd angle varies from *ca* 82 to 119° and the O—Cd⋯O angle from *ca* 60 to 90°.

## Synthesis and crystallization   

The synthesis of the ligand 5,6-bis­(pyridin-2-yl)pyrazine-2,3-di­carb­oxy­lic acid (**H_2_L**) has been reported previously (Alfonso *et al.*, 2001 ▸).

**Synthesis of the title coordination polymer**: **H_2_L** (32 mg, 0.10 mmol) was added to an aqueous solution (25 ml) of CdCl_2_·2H_2_O (22 mg, 0.10 mmol). The colourless solution immediately obtained was stirred for 1 h at room temperature. The reaction mixture was then filtered and the filtrate allowed to evaporate slowly at room temperature. After two weeks, small colourless plate-like crystals of the title compound were obtained, separated by filtration and dried in air (yield: 40 mg, 42.5%). Selected IR bands (KBr pellet, cm^−1^): ν 1630(*m*), 1598(*vs*), 1533(*m*), 1469(*m*), 1442(*m*), 1414(*m*), 1362(*s*), 1301(*m*), 1273(*m*), 1176(*m*), 1165(*m*), 1119(*m*), 1043(*w*), 992(*w*), 829(*m*), 789(*m*), 759(*m*), 675(*m*), 562(*m*), 513(*m*). Analysis for C_16_H_12_N_4_O_6_Cd (468.71): calculated: C 41.00, H 2.58, N 11.95%; found: C 40.70, H 2.43, N 11.80%.

## Refinement   

Crystal data, data collection and structure refinement details are summarized in Table 3 ▸. The water H atoms were located in a difference Fourier map and refined with distance restraints: O—H = 0.84 (2) and H⋯H = 1.35 (2) Å, with *U*_iso_(H) = 1.5*U*_eq_(O). The C-bound H atoms were included in calculated positions and treated as riding atoms: C—H = 0.94 Å with *U*_iso_(H) = 1.2*U*_eq_(C). The best crystal available was extremely thin (0.01 mm) and as the shape of the crystal was irregular it was not possible to carry out a numerical absorption correction. The displacement ellipsoids for two carboxyl­ate O atoms (O2 and O4) and a water O atom (O*W*1) are large but attempts to split these atoms were not successful.

## Supplementary Material

Crystal structure: contains datablock(s) I, Global. DOI: 10.1107/S2056989016012858/pk2589sup1.cif

Structure factors: contains datablock(s) I. DOI: 10.1107/S2056989016012858/pk2589Isup2.hkl

CCDC reference: 1498382

Additional supporting information: crystallographic information; 3D view; checkCIF report

## Figures and Tables

**Figure 3:**
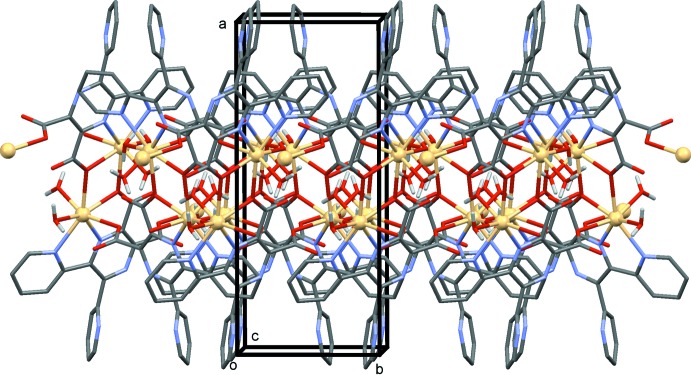
A view along the *c* axis of the title two-dimensional coordination polymer. The C-bound H atoms have been omitted for clarity.

**Table 1:** Selected geometric parameters (Å, °)

Cd1—O1	2.371 (4)	Cd1—N3	2.430 (4)
Cd1—O3^i^	2.377 (4)	Cd1—O1*W*	2.301 (4)
Cd1—N1	2.418 (4)	Cd1—O2*W*	2.317 (3)
Cd1—O1^ii^	2.427 (4)		
			
Cd1—O1—Cd1^ii^	107.74 (13)	O1*W*—Cd1—N1	91.62 (16)
O1*W*—Cd1—O3^i^	157.41 (15)	O1*W*—Cd1—N3	87.87 (15)
O1—Cd1—O1^ii^	72.26 (13)	O1*W*—Cd1—O1^ii^	76.59 (15)
O1—Cd1—N1	67.98 (13)	O2*W*—Cd1—O3^i^	87.05 (13)
N1—Cd1—N3	65.40 (14)	O1—Cd1—O3^i^	80.38 (12)
O2*W*—Cd1—N3	78.01 (13)	O3^i^—Cd1—N1	91.67 (13)
O2*W*—Cd1—O1^ii^	80.65 (13)	O3^i^—Cd1—O1^ii^	86.60 (12)
O1*W*—Cd1—O2*W*	104.67 (16)	O3^i^—Cd1—N3	113.74 (13)
O1*W*—Cd1—O1	80.18 (15)		

Symmetry codes: (i) 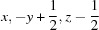; (ii) 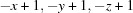.

**Table 2:** Hydrogen-bond geometry (Å, °)

*D*—H⋯*A*	*D*—H	H⋯*A*	*D*⋯*A*	*D*—H⋯*A*
O1*W*—H1*WA*⋯O3^iii^	0.82 (2)	2.22 (3)	2.974 (6)	152 (5)
O1*W*—H1*WB*⋯O2^iv^	0.84 (2)	2.05 (4)	2.805 (6)	150 (7)
O2*W*—H2*WA*⋯O4^i^	0.85 (2)	1.88 (3)	2.630 (6)	146 (5)
O2*W*—H2*WB*⋯O2^ii^	0.85 (2)	1.88 (2)	2.692 (5)	159 (5)
C9—H9⋯O3^v^	0.94	2.52	3.245 (6)	134
C14—H14⋯N4^vi^	0.94	2.62	3.372 (8)	137

Symmetry codes: (i) 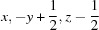; (ii) 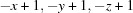; (iii) 
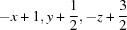
; (iv) 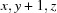; (v) 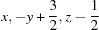; (vi) 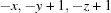.

**Table 3:** Experimental details

Crystal data	
Chemical formula	[Cd(C_16_H_8_N_4_O_4_)(H_2_O)_2_]
*M*_r_	468.70
Crystal system, space group	Monoclinic, *P*2_1_/*c*
Temperature (K)	223
*a*, *b*, *c* (Å)	16.6854 (12), 7.0799 (6), 13.4537 (10)
β (°)	96.236 (9)
*V* (Å^3^)	1579.9 (2)
*Z*	4
Radiation type	Mo *K*α
μ (mm^−1^)	1.43
Crystal size (mm)	0.30 × 0.20 × 0.01

Data collection	
Diffractometer	Stoe IPDS 1 image plate
Absorption correction	Multi-scan (*MULABS*; Spek, 2009 ▸)
*T*_min_, *T*_max_	0.900, 1.00
No. of measured, independent and observed [*I* > 2σ(*I*)] reflections	11782, 3056, 1781
*R*_int_	0.129
(sin θ/λ)_max_ (Å^−1^)	0.615

Refinement	
*R*[*F* ^2^ > 2σ(*F* ^2^)], *wR*(*F* ^2^), *S*	0.038, 0.063, 0.75
No. of reflections	3056
No. of parameters	257
No. of restraints	6
H-atom treatment	H atoms treated by a mixture of independent and constrained refinement
Δρ_max_, Δρ_min_ (e Å^−3^)	0.53, −0.59

Computer programs: *EXPOSE*, *CELL* and *INTEGRATE* in *IPDS-I* (Stoe & Cie, 2004 ▸), *SHELXS97* (Sheldrick, 2008 ▸), *SHELXL2014* (Sheldrick, 2015 ▸), *Mercury* (Macrae *et al.*, 2008 ▸), *PLATON* (Spek, 2009 ▸) and *publCIF* (Westrip, 2010 ▸).

## supplementary crystallographic information

### Crystal data

**Table:**

[Cd(C_16_H_8_N_4_O_4_)(H_2_O)_2_]	*F*(000) = 928
*M**_r_* = 468.70	*D*_x_ = 1.970 Mg m^−^^3^
Monoclinic, *P*2_1_/*c*	Mo *K*α radiation, λ = 0.71073 Å
*a* = 16.6854 (12) Å	Cell parameters from 5000 reflections
*b* = 7.0799 (6) Å	θ = 1.7–26.1°
*c* = 13.4537 (10) Å	µ = 1.43 mm^−^^1^
β = 96.236 (9)°	*T* = 223 K
*V* = 1579.9 (2) Å^3^	Plate, colourless
*Z* = 4	0.30 × 0.20 × 0.01 mm

### Data collection

**Table:**

Stoe IPDS 1 image plate diffractometer	3056 independent reflections
Radiation source: fine-focus sealed tube	1781 reflections with *I* > 2σ(*I*)
Plane graphite monochromator	*R*_int_ = 0.129
φ rotation scans	θ_max_ = 25.9°, θ_min_ = 2.5°
Absorption correction: multi-scan (MULABS; Spek, 2009)	*h* = −20→20
*T*_min_ = 0.900, *T*_max_ = 1.00	*k* = −8→8
11782 measured reflections	*l* = −16→16

### Refinement

**Table:**

Refinement on *F*^2^	Secondary atom site location: difference Fourier map
Least-squares matrix: full	Hydrogen site location: mixed
*R*[*F*^2^ > 2σ(*F*^2^)] = 0.038	H atoms treated by a mixture of independent and constrained refinement
*wR*(*F*^2^) = 0.063	*w* = 1/[σ^2^(*F*_o_^2^) + (0.0062*P*)^2^] where *P* = (*F*_o_^2^ + 2*F*_c_^2^)/3
*S* = 0.75	(Δ/σ)_max_ = 0.001
3056 reflections	Δρ_max_ = 0.53 e Å^−^^3^
257 parameters	Δρ_min_ = −0.59 e Å^−^^3^
6 restraints	Extinction correction: SHELXL2014 (Sheldrick, 2015), Fc^*^=kFc[1+0.001xFc^2^λ^3^/sin(2θ)]^-1/4^
Primary atom site location: structure-invariant direct methods	Extinction coefficient: 0.00055 (16)

### Special details

**Table:**

Geometry. All esds (except the esd in the dihedral angle between two l.s. planes) are estimated using the full covariance matrix. The cell esds are taken into account individually in the estimation of esds in distances, angles and torsion angles; correlations between esds in cell parameters are only used when they are defined by crystal symmetry. An approximate (isotropic) treatment of cell esds is used for estimating esds involving l.s. planes.

### Fractional atomic coordinates and isotropic or equivalent isotropic displacement parameters (Å^2^)

**Table:**

	*x*	*y*	*z*	*U*_iso_*/*U*_eq_	
Cd1	0.39911 (3)	0.63678 (6)	0.47689 (3)	0.01231 (13)	
O1	0.4663 (2)	0.3932 (6)	0.5741 (2)	0.0160 (9)	
O2	0.4579 (2)	0.1638 (6)	0.6875 (3)	0.0266 (10)	
O3	0.3636 (2)	0.1259 (6)	0.8712 (2)	0.0207 (9)	
O4	0.3010 (3)	−0.0413 (6)	0.7454 (3)	0.0436 (14)	
O1W	0.4770 (3)	0.8162 (6)	0.5928 (3)	0.0412 (13)	
H1WA	0.520 (2)	0.780 (9)	0.623 (4)	0.062*	
H1WB	0.457 (3)	0.898 (7)	0.628 (4)	0.062*	
O2W	0.3837 (2)	0.8219 (5)	0.3341 (2)	0.0189 (10)	
H2WA	0.355 (2)	0.766 (7)	0.287 (3)	0.028*	
H2WB	0.4295 (16)	0.833 (8)	0.312 (3)	0.028*	
N1	0.3193 (3)	0.5188 (6)	0.6031 (3)	0.0130 (10)	
N2	0.2284 (3)	0.3495 (7)	0.7347 (3)	0.0156 (10)	
N3	0.2853 (2)	0.8351 (6)	0.5067 (3)	0.0131 (10)	
N4	0.0603 (3)	0.5341 (6)	0.6152 (3)	0.0222 (12)	
C1	0.2472 (3)	0.5920 (7)	0.6161 (3)	0.0096 (12)	
C2	0.2003 (3)	0.4982 (7)	0.6816 (4)	0.0128 (13)	
C3	0.3478 (3)	0.3673 (9)	0.6546 (3)	0.0100 (10)	
C4	0.3025 (3)	0.2842 (7)	0.7250 (4)	0.0102 (13)	
C5	0.2290 (3)	0.7774 (7)	0.5650 (4)	0.0122 (13)	
C6	0.1657 (3)	0.8955 (8)	0.5818 (3)	0.0186 (14)	
H6	0.1260	0.8536	0.6211	0.022*	
C7	0.1608 (4)	1.0747 (7)	0.5410 (4)	0.0196 (14)	
H7	0.1174	1.1544	0.5516	0.024*	
C8	0.2194 (3)	1.1351 (9)	0.4850 (3)	0.0166 (11)	
H8	0.2175	1.2573	0.4576	0.020*	
C9	0.2815 (3)	1.0138 (7)	0.4696 (4)	0.0156 (13)	
H9	0.3224	1.0560	0.4322	0.019*	
C10	0.1146 (4)	0.5462 (7)	0.6958 (4)	0.0179 (14)	
C11	0.0946 (4)	0.5917 (8)	0.7907 (4)	0.0249 (15)	
H11	0.1347	0.6018	0.8453	0.030*	
C12	0.0141 (4)	0.6216 (10)	0.8025 (4)	0.0285 (14)	
H12	−0.0016	0.6492	0.8660	0.034*	
C13	−0.0426 (4)	0.6106 (9)	0.7204 (5)	0.0333 (16)	
H13	−0.0974	0.6328	0.7263	0.040*	
C14	−0.0169 (4)	0.5661 (8)	0.6298 (5)	0.0294 (17)	
H14	−0.0560	0.5574	0.5741	0.035*	
C15	0.4309 (3)	0.3025 (7)	0.6364 (4)	0.0140 (14)	
C16	0.3259 (3)	0.1061 (8)	0.7857 (4)	0.0146 (13)	

### Atomic displacement parameters (Å^2^)

**Table:**

	*U*^11^	*U*^22^	*U*^33^	*U*^12^	*U*^13^	*U*^23^
Cd1	0.0136 (2)	0.01131 (19)	0.01235 (19)	0.0013 (3)	0.00290 (13)	0.0012 (2)
O1	0.015 (2)	0.019 (2)	0.0148 (18)	−0.001 (2)	0.0050 (16)	0.0052 (18)
O2	0.023 (2)	0.026 (3)	0.034 (2)	0.013 (2)	0.0159 (18)	0.021 (2)
O3	0.032 (2)	0.019 (2)	0.0108 (18)	0.011 (2)	0.0008 (17)	−0.002 (2)
O4	0.064 (4)	0.015 (2)	0.042 (3)	−0.011 (2)	−0.039 (3)	0.003 (2)
O1W	0.029 (3)	0.032 (3)	0.057 (3)	0.010 (2)	−0.018 (2)	−0.031 (2)
O2W	0.017 (2)	0.023 (3)	0.017 (2)	−0.003 (2)	−0.0011 (17)	0.0062 (17)
N1	0.012 (3)	0.017 (3)	0.010 (2)	0.003 (2)	0.003 (2)	−0.0037 (19)
N2	0.018 (3)	0.015 (2)	0.014 (2)	0.005 (3)	−0.0004 (19)	0.002 (2)
N3	0.014 (3)	0.007 (3)	0.018 (2)	0.002 (2)	0.0006 (19)	0.0031 (19)
N4	0.012 (3)	0.027 (3)	0.028 (3)	0.004 (2)	0.004 (2)	0.002 (2)
C1	0.005 (3)	0.017 (3)	0.008 (2)	0.001 (2)	0.004 (2)	−0.002 (2)
C2	0.010 (3)	0.014 (3)	0.014 (3)	−0.007 (3)	−0.002 (2)	−0.002 (2)
C3	0.013 (3)	0.010 (2)	0.007 (2)	−0.004 (3)	0.001 (2)	0.001 (3)
C4	0.012 (3)	0.010 (3)	0.009 (3)	−0.005 (3)	0.002 (2)	−0.003 (2)
C5	0.008 (3)	0.017 (3)	0.013 (3)	0.000 (3)	0.004 (2)	−0.001 (2)
C6	0.019 (3)	0.021 (4)	0.016 (3)	0.004 (3)	0.007 (2)	0.003 (3)
C7	0.023 (4)	0.015 (3)	0.021 (3)	0.011 (3)	0.003 (3)	−0.004 (2)
C8	0.027 (3)	0.007 (2)	0.016 (3)	0.004 (3)	0.003 (2)	0.001 (3)
C9	0.020 (4)	0.017 (3)	0.011 (3)	−0.002 (3)	0.006 (3)	0.001 (2)
C10	0.020 (4)	0.014 (3)	0.020 (3)	0.002 (3)	0.003 (3)	0.006 (2)
C11	0.025 (4)	0.024 (4)	0.026 (3)	0.002 (3)	0.009 (3)	0.004 (3)
C12	0.032 (4)	0.021 (3)	0.037 (3)	0.003 (4)	0.021 (3)	0.004 (3)
C13	0.021 (4)	0.022 (4)	0.060 (4)	0.003 (4)	0.017 (3)	0.002 (4)
C14	0.013 (4)	0.028 (4)	0.045 (4)	0.003 (3)	−0.004 (3)	0.006 (3)
C15	0.015 (4)	0.015 (3)	0.012 (3)	−0.003 (3)	0.002 (3)	−0.006 (2)
C16	0.016 (3)	0.012 (3)	0.017 (3)	0.005 (3)	0.005 (2)	0.002 (3)

### Geometric parameters (Å, º)

**Table:**

Cd1—O1	2.371 (4)	N4—C14	1.344 (7)
Cd1—O3^i^	2.377 (4)	C1—C2	1.407 (7)
Cd1—N1	2.418 (4)	C1—C5	1.498 (7)
Cd1—O1^ii^	2.427 (4)	C2—C10	1.502 (8)
Cd1—N3	2.430 (4)	C3—C4	1.403 (7)
Cd1—O1W	2.301 (4)	C3—C15	1.506 (7)
Cd1—O2W	2.317 (3)	C4—C16	1.530 (7)
O1—C15	1.255 (6)	C5—C6	1.384 (7)
O1—Cd1^ii^	2.427 (3)	C6—C7	1.381 (7)
O2—C15	1.253 (6)	C6—H6	0.9400
O3—C16	1.258 (6)	C7—C8	1.366 (7)
O3—Cd1^iii^	2.377 (4)	C7—H7	0.9400
O4—C16	1.227 (6)	C8—C9	1.378 (7)
O1W—H1WA	0.82 (2)	C8—H8	0.9400
O1W—H1WB	0.837 (19)	C9—H9	0.9400
O2W—H2WA	0.847 (19)	C10—C11	1.392 (7)
O2W—H2WB	0.852 (19)	C11—C12	1.386 (8)
N1—C3	1.335 (7)	C11—H11	0.9400
N1—C1	1.339 (6)	C12—C13	1.376 (8)
N2—C2	1.328 (7)	C12—H12	0.9400
N2—C4	1.341 (7)	C13—C14	1.373 (8)
N3—C5	1.351 (6)	C13—H13	0.9400
N3—C9	1.359 (6)	C14—H14	0.9400
N4—C10	1.338 (7)		
			
Cd1—O1—Cd1^ii^	107.74 (13)	C1—C2—C10	125.1 (5)
O1W—Cd1—O3^i^	157.41 (15)	N1—C3—C4	120.0 (5)
O1—Cd1—O1^ii^	72.26 (13)	N1—C3—C15	116.3 (4)
O1—Cd1—N1	67.98 (13)	C4—C3—C15	123.6 (5)
N1—Cd1—N3	65.40 (14)	N2—C4—C3	119.4 (5)
O2W—Cd1—N3	78.01 (13)	N2—C4—C16	114.6 (4)
O2W—Cd1—O1^ii^	80.65 (13)	C3—C4—C16	125.6 (5)
O1W—Cd1—O2W	104.67 (16)	N3—C5—C6	120.2 (5)
O1W—Cd1—O1	80.18 (15)	N3—C5—C1	114.3 (5)
O1W—Cd1—N1	91.62 (16)	C6—C5—C1	125.1 (5)
O1W—Cd1—N3	87.87 (15)	C7—C6—C5	120.3 (5)
O1W—Cd1—O1^ii^	76.59 (15)	C7—C6—H6	119.9
O2W—Cd1—O3^i^	87.05 (13)	C5—C6—H6	119.9
O1—Cd1—O3^i^	80.38 (12)	C8—C7—C6	119.4 (5)
O3^i^—Cd1—N1	91.67 (13)	C8—C7—H7	120.3
O3^i^—Cd1—O1^ii^	86.60 (12)	C6—C7—H7	120.3
O3^i^—Cd1—N3	113.74 (13)	C7—C8—C9	118.9 (5)
O2W—Cd1—N1	139.31 (15)	C7—C8—H8	120.6
O2W—Cd1—O1	150.65 (13)	C9—C8—H8	120.6
N1—Cd1—O1^ii^	139.91 (14)	N3—C9—C8	122.1 (5)
O1—Cd1—N3	131.34 (12)	N3—C9—H9	119.0
O1^ii^—Cd1—N3	149.40 (14)	C8—C9—H9	119.0
C15—O1—Cd1	120.8 (3)	N4—C10—C11	123.3 (5)
C15—O1—Cd1^ii^	131.4 (4)	N4—C10—C2	116.9 (5)
C16—O3—Cd1^iii^	121.8 (4)	C11—C10—C2	119.7 (5)
Cd1—O1W—H1WA	124 (4)	C12—C11—C10	118.2 (6)
Cd1—O1W—H1WB	122 (4)	C12—C11—H11	120.9
H1WA—O1W—H1WB	109 (3)	C10—C11—H11	120.9
Cd1—O2W—H2WA	111 (4)	C13—C12—C11	119.3 (5)
Cd1—O2W—H2WB	109 (4)	C13—C12—H12	120.4
H2WA—O2W—H2WB	104 (3)	C11—C12—H12	120.4
C3—N1—C1	121.2 (4)	C14—C13—C12	118.2 (6)
C3—N1—Cd1	116.7 (3)	C14—C13—H13	120.9
C1—N1—Cd1	121.9 (3)	C12—C13—H13	120.9
C2—N2—C4	119.7 (4)	N4—C14—C13	124.4 (6)
C5—N3—C9	119.1 (4)	N4—C14—H14	117.8
C5—N3—Cd1	121.8 (3)	C13—C14—H14	117.8
C9—N3—Cd1	118.9 (3)	O2—C15—O1	126.9 (5)
C10—N4—C14	116.5 (5)	O2—C15—C3	115.6 (5)
N1—C1—C2	117.9 (5)	O1—C15—C3	117.5 (5)
N1—C1—C5	114.8 (4)	O4—C16—O3	127.6 (5)
C2—C1—C5	127.0 (5)	O4—C16—C4	114.3 (5)
N2—C2—C1	121.6 (5)	O3—C16—C4	118.0 (5)
N2—C2—C10	113.3 (4)		
			
C3—N1—C1—C2	−3.4 (7)	C6—C7—C8—C9	1.0 (8)
Cd1—N1—C1—C2	171.5 (3)	C5—N3—C9—C8	−3.6 (7)
C3—N1—C1—C5	171.2 (4)	Cd1—N3—C9—C8	−178.9 (4)
Cd1—N1—C1—C5	−13.9 (6)	C7—C8—C9—N3	1.2 (8)
C4—N2—C2—C1	−1.1 (8)	C14—N4—C10—C11	−0.9 (8)
C4—N2—C2—C10	176.2 (5)	C14—N4—C10—C2	176.1 (5)
N1—C1—C2—N2	4.7 (7)	N2—C2—C10—N4	−117.2 (5)
C5—C1—C2—N2	−169.2 (5)	C1—C2—C10—N4	60.0 (7)
N1—C1—C2—C10	−172.3 (5)	N2—C2—C10—C11	59.9 (7)
C5—C1—C2—C10	13.8 (8)	C1—C2—C10—C11	−122.9 (6)
C1—N1—C3—C4	−1.2 (7)	N4—C10—C11—C12	1.5 (8)
Cd1—N1—C3—C4	−176.3 (4)	C2—C10—C11—C12	−175.3 (5)
C1—N1—C3—C15	−177.7 (4)	C10—C11—C12—C13	−1.7 (9)
Cd1—N1—C3—C15	7.1 (5)	C11—C12—C13—C14	1.3 (10)
C2—N2—C4—C3	−3.6 (7)	C10—N4—C14—C13	0.4 (9)
C2—N2—C4—C16	−176.7 (5)	C12—C13—C14—N4	−0.7 (10)
N1—C3—C4—N2	4.8 (8)	Cd1—O1—C15—O2	175.2 (4)
C15—C3—C4—N2	−178.9 (5)	Cd1^ii^—O1—C15—O2	−2.5 (8)
N1—C3—C4—C16	177.2 (5)	Cd1—O1—C15—C3	−6.1 (6)
C15—C3—C4—C16	−6.5 (8)	Cd1^ii^—O1—C15—C3	176.2 (3)
C9—N3—C5—C6	3.7 (7)	N1—C3—C15—O2	177.9 (4)
Cd1—N3—C5—C6	178.9 (4)	C4—C3—C15—O2	1.5 (8)
C9—N3—C5—C1	−169.7 (4)	N1—C3—C15—O1	−0.9 (7)
Cd1—N3—C5—C1	5.5 (6)	C4—C3—C15—O1	−177.3 (5)
N1—C1—C5—N3	5.2 (6)	Cd1^iii^—O3—C16—O4	1.6 (8)
C2—C1—C5—N3	179.3 (5)	Cd1^iii^—O3—C16—C4	177.4 (3)
N1—C1—C5—C6	−167.8 (5)	N2—C4—C16—O4	82.7 (6)
C2—C1—C5—C6	6.3 (9)	C3—C4—C16—O4	−90.0 (7)
N3—C5—C6—C7	−1.5 (8)	N2—C4—C16—O3	−93.7 (6)
C1—C5—C6—C7	171.1 (5)	C3—C4—C16—O3	93.6 (6)
C5—C6—C7—C8	−0.8 (8)		

Symmetry codes: (i) *x*, −*y*+1/2, *z*−1/2; (ii) −*x*+1, −*y*+1, −*z*+1; (iii) *x*, −*y*+1/2, *z*+1/2.

### Hydrogen-bond geometry (Å, º)

**Table:**

*D*—H···*A*	*D*—H	H···*A*	*D*···*A*	*D*—H···*A*
O1*W*—H1*WA*···O3^iv^	0.82 (2)	2.22 (3)	2.974 (6)	152 (5)
O1*W*—H1*WB*···O2^v^	0.84 (2)	2.05 (4)	2.805 (6)	150 (7)
O2*W*—H2*WA*···O4^i^	0.85 (2)	1.88 (3)	2.630 (6)	146 (5)
O2*W*—H2*WB*···O2^ii^	0.85 (2)	1.88 (2)	2.692 (5)	159 (5)
C9—H9···O3^vi^	0.94	2.52	3.245 (6)	134
C14—H14···N4^vii^	0.94	2.62	3.372 (8)	137

Symmetry codes: (i) *x*, −*y*+1/2, *z*−1/2; (ii) −*x*+1, −*y*+1, −*z*+1; (iv) −*x*+1, *y*+1/2, −*z*+3/2; (v) *x*, *y*+1, *z*; (vi) *x*, −*y*+3/2, *z*−1/2; (vii) −*x*, −*y*+1, −*z*+1.
